# Identifying priorities, directions and a vision for Indigenous mental health using a collaborative and consensus-based facilitation approach

**DOI:** 10.1186/s12913-022-07682-3

**Published:** 2022-03-27

**Authors:** Stephanie Montesanti, Kayla Fitzpatrick, Bryan Fayant, Caillie Pritchard

**Affiliations:** 1 School of Public Health, University of Alberta, 3-300 Edmonton Clinic Health Academy, 11405 - 87 Ave, Edmonton, AB T6G 1C9 Canada; 2 McMurray Metis, 441 Sakitawaw Trail, Fort McMurray, Alberta, T9H 4P3 Canada

**Keywords:** Indigenous mental health, Holistic mental health service delivery, Cultural safety, Integrated models of care, Cross-sectoral collaboration, Community visioning, Priority setting, Nominal group technique consensus method, Children and youth

## Abstract

**Background:**

Mental health disparities between Indigenous and non-Indigenous people in Canada are related to underlying economic, social, and political inequities that are legacies of colonization and the oppression of Indigenous cultures. It also widely acknowledged that mental health services currently available may not be culturally appropriate in supporting the health needs of Indigenous Canadians. A two-day Indigenous mental health forum examined mental health needs and gaps among Indigenous communities across the Regional Municipality of Wood Buffalo (RMWB) on Treaty 8 territory, in northern Alberta, Canada. This paper outlines the insights generated by stakeholder engagement at the forum to identify and prioritize directions for Indigenous mental health and build a vision and strategy for improving mental health services and programs for the region’s diverse Indigenous population.

**Methods:**

We applied a modified nominal group technique (NGT) consensus method embedded within Indigenous knowledge to determine key priorities and directions for Indigenous-focused mental health and synthesize information from discussions that occurred at the forum. Following the NGT, a participatory community visioning exercise was conducted with participants to develop a vision, guiding principles, and components of an action plan for an Indigenous mental health strategy for the RMWB.

**Results:**

Four key themes for setting priorities and directions for Indigenous mental health emerged from roundtable group discussions: 1) understand the realities of mental health experiences for Indigenous peoples, 2) design a holistic and culturally rooted mental health system, 3) foster cross-sectoral engagement and collaboration on mental health service delivery, and 4) focus on children and youth. The community visioning exercise helped stakeholders to visualize a direction or path forward for addressing existing gaps in the mental health system and opportunities for strengthening Indigenous mental health in the region.

**Conclusions:**

Forum participants described mental health and well-being around holistic concepts of social and emotional well-being. Addressing Indigenous mental health and wellness involves multi-sectoral action in various settings including community and school through programs, policies, and other interventions that promote mental health for all Indigenous peoples, as well as for those at greater risk such as children and youth.

**Supplementary Information:**

The online version contains supplementary material available at 10.1186/s12913-022-07682-3.

## Introduction

Mental health problems, indicated by outcomes such as suicide and emotional distress, are generally higher among Indigenous peoples in Canada. These disparities in mental health relate to underlying economic, social, and political inequities that are legacies of colonization and the Canadian government’s attempt at cultural genocide [1]. During the COVID-19 pandemic, Indigenous young people, families, and communities within Canada have faced new and pre-existing challenges to their mental health. Indigenous people possess a wealth of diverse healing traditions for promoting positive mental health and emotional well-being that have endured despite cultural oppression of colonization. However, connection to these traditions has been affected during the COVID-19 pandemic due to public health protocols, lack of ability to travel to communities due to lockdown measures, and the shutdown of cultural gatherings, adding further stress on families and entire communities [2]. Heck et al*.* (2021) described how “physical distancing makes it difficult to interact with people who are at risk of experiencing mental health challenges and to organize ways to collectively grieve and heal” [3]. Indigenous peoples have expressed the desire for access to traditional ceremonies and medicines to discover solutions and help cope with pandemic challenges [4].

To address the mental health disparities that exist within Indigenous populations, effective components of mental health programs and services for Indigenous peoples need to be identified [5]. Given the historical and intergenerational trauma brought on by settler colonialism, mental health programs and services designed and led by Indigenous people and communities hold greater promise at improving mental health and well-being compared to western-based approaches [6, 7]. Furthermore, the need to address the growing mental health disparities in Indigenous peoples compared with non-Indigenous peoples in Canada was emphasized in the findings of the Truth and Reconciliation Commission of Canada (TRC) [1]. The TRC’s Calls to Action on health envision how health equity might be achieved through transforming mental health services for Indigenous peoples in Canada [8].

Mental health promotion “involves actions to create living conditions and environments that support mental wellness across the lifespan and allow people to adopt and maintain healthy lifestyles” [9]. This requires cross-sectoral action across home, school, health services and community environments, through culturally safe, strengths-based, family- and community-oriented mental health promotion programs, services, and policies that promote healthy emotional, spiritual, and social development in childhood and adolescence, as well as for those at risk of poor mental health [10]. Despite calls of integrated approaches to address mental health problems and illnesses among Indigenous peoples, we know little about how services work together cross-sectorally to ensure communities receive a continuum of preventative and curative services, according to their needs and priorities, and across different levels of the health and social system [10].

In this paper we report on the findings from an adapted nominal group technique (NGT) consensus [11, 12] and community visioning exercise [13] with a diverse group of cross-sectoral stakeholders, including community members, in the Regional Municipality of Wood Buffalo (RMWB) located in northern Alberta, Canada. We held a two-day Indigenous mental health forum to identify and prioritize directions for Indigenous mental health and build a vision and strategy for improving coordinated and integrated mental health services and programs for Indigenous populations in the RMWB. Both the NGT and community visioning methods have been adapted to support group decision-making and priority-setting with Indigenous participants in the areas of health and community development [14, 15]. The forum’s aims were to: 1) discuss the challenges and opportunities in addressing mental health among Indigenous youth, adults, and families; 2) network key stakeholders across the region’s sectors to explore where sectors could overlap and collaborate on improving mental health services for Indigenous populations; and 3) create innovative recommendations to support mental health wellness for Indigenous populations across the life course. We followed a similar approach as the one conducted by Chatwood et al*.* (2015) by applying a mixed methods approach with western-based research methodologies (i.e., NGT and community visioning) and the incorporation of Indigenous knowledge through storytelling and dialogue [15]. This paper contributes valuable insights to applying collaborative and consensus-based facilitation approaches to strengthen cross-sectoral engagement for Indigenous mental health.

### Literature review: Indigenous peoples’ historical and contemporary experiences with access to mental healthcare in Canada

The United Nations recognizes Indigenous peoples as those who self-identify and are accepted by their community as their member, with this community having historical continuity to precolonial and/or presettler societies, with distinct cultural practices and social, economic, and political systems [16]. The mental health status of Indigenous peoples in Canada must be understood within the context of current and historical colonial experiences, from the loss of land and autonomy to the creation of the reserves systems, the historical removal of Indigenous children into residential schools, the current removal of Indigenous children by the child welfare system, and systemic and epistemic racism in healthcare settings [17, 18].

Inequitable access to healthcare in Canada is influenced by the complexity of multiple governing bodies overseeing healthcare delivery, with lack of jurisdictional coordination leading to gaps in care and disparities in funding and data collection within Indigenous populations [19, 20]. Remote communities face a lack of service providers, lack of health infrastructure and services, and high costs [20]. While lack of a sufficient number of mental health professionals is a concern across Canada [21], accessibility and availability of mental healthcare is particularly lacking for Indigenous populations, especially those in rural, remote, and northern settings. Scholars have pointed to the absence of a national Indigenous mental health strategy and subsequent inadequate funding as barriers to both recruiting mental health professionals in rural and remote Indigenous communities and to providing culturally competent mental health services [22]. Additionally, different Indigenous groups in Canada, such as Métis [19, 23] and urban or off-reserve First Nation communities, can have unique experiences that are often excluded in research and data, and which may require different culturally based responses. Indigenous people also face many structural barriers due to the legacy of colonialism in a healthcare system where western knowledge is valued over Indigenous knowledge, trauma-informed care is not consistently practiced, and negative bias from care providers persists due to lack of cultural competency [19, 24–26]. Given the context of historical trauma and ongoing discrimination faced by Indigenous people, culturally appropriate mental health services are imperative [20, 27]. Moreover, self-determination, defined as the individual and collective right to have control over health, education, and economic systems, is a key determinant of health for Indigenous individuals and communities [28]. In this sense, self-determination shapes healthcare experiences for Indigenous peoples.

Many Indigenous communities’ perspectives on health are holistic and centred on a connection to culture [29, 30]. Research has supported the importance of mental health clinicians practicing multicultural competence when working with Indigenous peoples, which includes acknowledging and understanding the social and cultural realities of Indigenous peoples and historical impacts of colonization [31]. The strengthening of cultural and community connectedness has been recommended for improving mental health services provided to Indigenous peoples [31]. Consultation with Elders by Drost [32] provided perspectives on how Alberta Health Services (AHS)—a provincial health authority in Canada — can expand their traditional Indigenous healing practices, which included enhancing cultural competency training for staff, and creating and maintaining partnerships with Indigenous communities.

The use of collaboration and teamwork has also been proposed to improve mental health services for Indigenous peoples. While the integration of Indigenous Elders within mental health services was suggested in the TRC, few health systems have attempted to integrate this, though some research initiatives have noted preliminary success [33, 34]. Restoule et al*.* (2016) emphasized the need to build partnerships between governments and Indigenous communities in order to continue building mental wellness, and supported the creation of multidisciplinary Mental Wellness Teams that included Elders, community workers, social workers, psychiatrists, and other health professionals [30]. However, more research is needed to understand opportunities and barriers for Indigenous health practitioners [24], and to fill the gap in knowledge regarding integration of Indigenous mental health workers and Indigenous Elders to provide mental health care. In order to truly have culturally appropriate mental health programs and policies [23, 35], Indigenous communities need to be involved in research and consulted throughout policy-making, and receive adequate funding for mental health services. Moreover, needed transformations identified in the TRC Calls to Action for mental health include: 1) eliminating health care resource disparities; 2) ensuring culturally safe services free of racism; 3) building provider capacity to effectively support healing by addressing impacts from multigenerational adverse life experiences; and 4) engaging Indigenous people within systems so that inclusion of diverse Indigenous cultures and wellness practices may promote optimal outcomes [8].

### Setting

The RMWB is located in a northern region within the Canadian province of Alberta and on Treaty 8 Traditional Territory, which encompasses a landmass of approximately 840,000 square kilometres and is home to 39 First Nations communities across the provinces of Alberta, Saskatchewan, British Columbia, and the Northwestern Territories. The RMWB is home to five First Nation communities (Mikisew Cree First Nation, Athabasca Chipewyan First Nation, Fort McKay First Nation, Fort McMurray First Nation, Chipewyan Prairie Dene First Nation), and lies within the Métis Nation of Alberta (MNA) Regions 1 and 5, which includes five Métis Locals located in Fort McMurray, Anzac, Fort Chipewyan, Fort McKay, and Conklin.

Indigenous peoples residing in the RMWB experienced one of the largest wildfire evacuations in Canadian history in 2016 (known as the Horse River wildfire) which resulted in community destruction and displacement, loss of homes, jobs, finances, lives as well as injuries and separation from loved ones. Five years post-wildfire, many individuals continue to be impacted by the social, emotional, and psychological difficulties posed by the wildfire. The COVID-19 pandemic in the region has posed a disproportionate threat to Indigenous communities, due to disparities in known risk factors for poorer COVID-19-related outcomes such as chronic health conditions, inadequate healthcare service/infrastructure, and limited resources (e.g., running water, housing) [36]. These disparities add to heightened vulnerabilities for mental health distress [37].

## Methods and approach

### Modified NGT consensus

The forum adopted an NGT, a structured method used for generating ideas and identifying solutions within groups, with the intention of generating priorities and directions for Indigenous mental health in the region. The method involves interactive group discussions to reach consensus and as such has the benefit of generating qualitative data to garner rich accounts of perspectives on a given topic [15, 38]. This method has been successfully used for gathering important input into evidence-informed policy and practice in the mental health field [39]. We used the NGT method instead of other consensus methods (i.e., a Delphi expert consensus which relies on feedback through the use of iterative questionnaires) for several reasons. Firstly, involving community service providers and mental health professionals in a face-to-face structured meeting enabled first-hand information to be obtained from those working with communities, families, and youth to support mental health. Secondly, the approach encourages participation from diverse cross-sectoral stakeholders. Thirdly, it is time efficient [40] being a single-occasion process and reducing the time burden on stakeholders, while also acquiring a substantial amount of information in a relatively short time. Fourthly, it reduces the influence of response bias resulting from intergroup dynamics and the researchers’ presence [41]. Lastly, NGT allows for in-session completion and immediate dissemination of results to the group. The NGT method also served as a stakeholder engagement strategy to support the co-design of priorities and directions for Indigenous mental health and build relationships [42]. Our modified NGT method involved: (1) the presentation of research evidence on Indigenous mental health outcomes and holistic frameworks for guiding Indigenous mental health interventions and program delivery; (2) four roundtable group discussions where participants generated and shared ideas relating to specific questions (see below); (3) individual voting on priorities for Indigenous mental health using a Dotmocracy method [43]; and (4) group discussion about the voting of priorities.

Qualitative research methods considered to be well aligned with Indigenous health research include consensus methods [15, 44]. The nominal group process also employed an Indigenous framework of Two-Eyed Seeing [45, 46] for equitably embracing multiple perspectives. Two-Eyed Seeing is defined as “learning to see from one eye with the strengths of Indigenous knowledges and ways of knowing, and from the other eye with the strengths of mainstream knowledges and ways of knowing, and to use both these eyes together, for the benefit of all” [46]. While the nominal consensus method is a western paradigm, it has been suggested that it can be responsive to Indigenous knowledge and allows space to share a variety of perspectives to reach consensus on the topic in question [15, 47]. Moreover, the anonymity, autonomy, and relational nature of collaborative and consensus-based techniques like the NGT [48] is compatible with Indigenous research principles (relationality, reciprocity, and respect) [15, 44, 49, 50].

We followed the recommendations by Humphrey-Murto et al. (2016) to ensure methodological rigour when using consensus group methods [51]. The NGT has demonstrated validity in enabling consensus on complex issues while considering participants’ views equally [52]. Scholars have concluded that the validity and effectiveness of the NGT is reflected in the level of expertise of the participants [53]. van Teijlingen et al. (2006) reviewed the merits and applications of using NGT to explore and collate expert opinion and concluded that it has validity, provided the facilitator does not attempt to overcome diversity of opinion to create artificial consensus [54, 55]. We also adhered to strategies for ensuring validity in qualitative research by acknowledging the researcher’s perspective, providing thick descriptions of participant responses, and methodological triangulation (e.g., western methods (qualitative) and Indigenous knowledge). Thus, in bringing together a diverse group of individuals from different sectors and professional settings, our forum provided a unique and valuable opportunity for mutual understanding and clarification of issues related to Indigenous mental health in the region.

### Participant recruitment

Invitation letters to key stakeholders to participate in the two-day forum were sent by email. Representation at the forum spanned across multiple sectors to include Indigenous leadership, community service providers, mental health professionals, schoolteachers, representatives from the Fort McMurray School District, Indigenous Elders, youth residing in the urban and rural communities, representatives from provincial and local organizations with mandates for Indigenous Health and Wellness, and non-Indigenous health care, academic, and research organizations. In total, fifty-three (*N* = 53) stakeholders (Table 1) attended the forum, which included all members of the Community Advisory Committee that guided preparation of the forum (as described below). While using NGT to prioritize issues may be difficult with a higher number of participants and groups, it has been shown to be successful in several studies [56, 57].

**Table 1:** Participating organizations

Provincial and local organizations with mandates for Indigenous Health and Wellness	Alberta Health Services (Wellness and Recovery Services)
	Alberta Health Services (Community Wellness Travelling Team)
	McMurray Métis Local 1935
	Nistawoyou Friendship Centre
	Conklin Community Association
	Nunee Health Board
	Chipewyan Prairie Dene Health Services
	Janvier Dene Wood Buffalo Community Association
	Critical Incident Stress Management
	Waypoints
	YMCA of Wood Buffalo
	Fort McMurray Public School District
	Fort McMurray Catholic Board of Education
	Dr. Clark School
	Borealis Counselling Services
	Regional Municipality of Wood Buffalo (Indigenous & Rural Relations)
	Canadian Red Cross
Non-Indigenous health care organizations	Alberta Health
	Alberta Health Services (Mental Health Promotion & Illness Prevention Team)
Academic and research organizations	The University of Alberta

### Roundtable group discussions

Participants were invited to generate and prioritize key directions for Indigenous mental health for the region in roundtable groups. Roundtable group discussions were guided by the following questions:

- 1. What matters most to you in promoting mental wellness for Indigenous youth, families, or communities?
- 2. What mental health resources are critical to advocate for in your community or within your organization?
- 3. How do current mental health services or programs address the root social causes (e.g., poverty, racism, housing, geography, etc.) of poor mental health? How might services or programs be strengthened for Indigenous clients?
- 4. How can the different sectors (e.g., health, education, justice) collaborate to support integrated and coordinated mental health and wellness among Indigenous peoples?

On day one of the forum, the organizers (SM, BF) described the objectives of the two-day forum. Participants were divided into four roundtable groups, lasting an average of 60 min and ranging from thirteen to fourteen participants, to explore each question with a discussion facilitator who resided in the region. Participants had the opportunity to rotate and contribute to discussions for all four questions. The first author (SM) developed a facilitation guide for roundtable group facilitators. NGT is an approach to group dialogue and decision-making that places weight on all participants having an equal opportunity to express a view. It is an effective way to enable people who might otherwise be excluded from decision-making to contribute [58]. As the first step, participants were invited to silently reflect and generate responses on provided cue cards following the facilitators’ posing of the question. For the second step, all participants were invited to share, discuss, and explore their proposed directions. Facilitators employed both open discussion and talking circle approaches to ensure all participants had opportunity for input. With permission from participants, conversations were audio-recorded and transcribed and detailed notes were taken by facilitators. At the end of day one, facilitators reported back to the larger group on key insights that were shared at their groups. Following each roundtable discussion, facilitators collected all cue cards from participants, sorted them according to emerging themes, and displayed the cue cards by theme on the wall. At the end of day one, facilitators reported back to the larger group key insights that were shared at their groups.

On day two, participants reviewed, reflected, and voted on the priorities and directions they felt were the highest priority for each of the cue cards displayed by theme on the wall. A modified Dotmocracy method was facilitated by the lead author (SM) as a means of promoting transparency of idea sharing and ranking, and of being inclusive of diverse stakeholder voices. Participants were provided red stickers for each display area to cast their votes. Red stickers were placed next to ideas that they felt were priorities. Dotmocracy is a consensus-building method that uses a democratic voting procedure to provide a structured approach to decision-making [43]. The method promotes transparency by allowing participants to observe the development of priorities around a topic or issue [59] and is inclusive of diverse stakeholder voices.

### Community visioning

Following the Dotmocracy exercise, participants gathered in a large group to co-create a community vision for developing an Indigenous mental health strategy for the RMWB (See Additional File 1 for a graphic recording on community vision). The community vision was developed using a participatory community visioning exercise [60, 61] with three parts: a) developing the vision, b) defining the guiding principles, and c) identifying strengths and the components of an action plan to develop an Indigenous mental health strategy. Participatory community visioning is a method employed within action research [62] and can assist in generating “integrative conceptions of place and supporting arguments” and facilitate “a degree of mutual understanding and even ownership among the stakeholders” [61, 63]. The visioning exercise helped stakeholders to visualize a direction or path forward for addressing existing gaps in the mental health system and opportunities for strengthening Indigenous mental health in the region.

#### Community advisory committee

We followed a community-based participatory research (CBPR) approach [64] to support engagement with communities, health professionals, and leaders across the sectors in the RMWB. The objectives and plan for the forum were developed in partnership with a Community Advisory Committee. The committee was comprised of six advisory members, including two Indigenous health service providers, one non-Indigenous health service provider, as well Indigenous Elders or Knowledge Holders from First Nation and Métis ancestry residing in the RMWB (one Dene-speaking Elder, one Cree-speaking Elder, and one Métis Knowledge Holder). The research team engaged the Community Advisory Committee in identifying the objectives and purpose of the forum, the roundtable group discussion questions, and the facilitation and dialogue approach to ensure that Indigenous knowledge was valued and to balance power relationships among community, service providers, and researchers in roundtable discussions [65, 66]. The committee also provided guidance on which key stakeholders to invite across the sectors, as well as ensuring community representation.

#### Data analysis

The final ranking of priorities from the Dotmocracy method was analyzed in relation to the notes taken by group facilitators to produce a consolidated list of key priorities and directions for Indigenous mental health for the region. The analysis of the roundtable discussions went beyond the presentation of the ranked list of priorities and directions to thematically analyze the roundtable group discussions. The four roundtable group discussions were transcribed verbatim by an external professional company and imported into NVivo 12 (QSR International) for analysis. Our thematic analysis was an iterative process that consisted of three stages involving inductive analysis. The initial analysis was initiated by the roundtable group facilitators who organized the cue cards into broad high-level themes. Next, the lead author (SM) reviewed the recordings and transcripts from the roundtable group discussions and the preliminary themes to develop a framework for priorities and directions for Indigenous mental health for the region (see Tables 2 3, 4, 5). Inductive analysis involved close coding to identify themes emerging from the data for each roundtable group question, and then cross-referencing of themes across all four data transcripts. At the third stage, the research team (SM, KF) and community partner (BF) met to refine key themes, sub-themes, and the nature and extent of their interconnectedness to inform priorities and direction for Indigenous mental health for the region. Quotes from participants were extracted from the transcripts to help explain both individual and group thinking. The final stage of the process was a presentation of the analysis and the framework to the Community Advisory Committee for feedback and confirmation of the key themes.

**Table 2:** Understand the realities of Indigenous mental health experience (Theme 1)

Realities of Mental Health Experience			
**Gaps**	**Education**	**Destigmatizing Mental Health**	**Indigenous Voice**
• Lack of consistency and availability of mental health services. Increase frequency of services and supports for mental health (i.e., services and supports should be available more than once a week) • Lack of reliable transportation between rural and to urban communities. Include transportation for residents living in rural and remote communities to access mental health services in urban centres • Services and support programs need to be offered in the Indigenous languages (i.e., Cree, Dene, Michif) • Service providers such as mental health therapists who travel from urban centres to rural communities have limited time to spend with clients. Additional funding is needed to hire mental health professionals that provide care in rural and remote communities	• Mental health providers need to understand the history of Indigenous peoples and the impacts from colonialism on intergenerational trauma • Effective mental health care for Indigenous patients is shaped by a deep understanding of the causal relationships between social factors specific to Indigenous people and health • Land-based learning and exploring the lived experience of Indigenous peoples are ways to help providers to understand, acknowledge, and identify multigenerational adverse life experiences •Mental health professionals need to build relationships and trust with their Indigenous clients. Spending more time with their clients can allow providers to explore and understand the root social causes of inequities in health	• Empower individuals to understand mental health and wellness without having the stigma associated with mental illness • Change the way we talk about and view mental illness and health	• Community needs to be engaged in the design of mental health programs and services • Create opportunities for Indigenous communities to influence the mental health service delivery model • Community drives the focus of mental wellness, not government-initiated programs and services • Engage the communities to understand what healing and mental wellness means to them. “Nothing about us without us”
**Participant Quotes**			
“Consistency is having the same person come in and not different service organizations …because you open up once, you want to keep talking to that same person. You don’t want to have to keep telling your story over and over again…”	“The workers need to build up the trust and rapport if they’re going to work in the community. Because they [referring to Indigenous residents] are not going to come to you. The workers are the ones that got to build that relationship.”	“Maybe we need to start talking about mental health differently and talking about brain health, so that we start to change the image of it as something that is broken and needs treatment.”	“We really need a holistic, coordinated approach that’s coming from our community members, not from me as a service provider, or a community leader, but truly at the heart of the grassroots level from our members.”

**Table 3:** Design a holistic and culturally rooted mental health system (Theme 2)

Holistic and Culturally Rooted Mental Health System			
**Hiring and Training Indigenous Mental Health Workers**	**Engage Elders**	**Cultural Appropriateness**	**Trauma-Informed Care**
• Support capacity building opportunities within the community. Allocate funding to train and hire Indigenous mental health workers or Indigenous mental health navigators from the communities who can provide cultural supports and traditional healing to Indigenous residents • Employing people in the community builds local capacity	• Engage Elders and Knowledge Holders from the communities as ‘cultural mentors’ to be involved in the training and education of mental health professionals • Engage Elders in the Schools to teach youth about cultural and traditional practices • Hire Elders to work alongside health professionals by participating in home visits	• Move away from the medical model of wellness/sickness to include Indigenous ways of knowledge on health and healing (i.e., use the Medicine Wheel) • Reclaim traditional healing practices such as native counselling and offering traditional foods (not market-based/western foods) in the communities • Focus on the ‘whole person’ (individual, family, and community needs) to promote healing • Promote cultural sensitivity across the spectrum of care, from prevention to promotion of mental health	• Embed Indigenous culture and values into healing and treatments for mental distress • Addiction and counselling services should be offered across the individual’s life span • Acknowledging and implementing the TRC recommendations across organizations
**Participant Quotes**			
“Staff or provider turn-over is a big challenge. Providers outside of the community are going to invest very differently in a community when they don’t expect to stay, because they don’t put down their roots [in the community].” “Community-led, community-run facilities are needed to address mental health. We are always going to encounter turn-over and lack of trust from community members [of providers] if we don’t have these services and programs embedded in our communities.” “And we are building the capacity of our members and our residents who are there because they do have the knowledge, they have the connections and their relationships, and they do have their hearts in the community.”	“…and that means moving away from mainstream education simply in your classroom…and going back to our Elders, and whether that’s involving Elders in a sort of formal education system or if that’s Elders educating professionals, I don’t know. We need to do that, but I think we need to look to our Elders who have that knowledge.”	“In order for the health system to better meet the needs of Indigenous people, first of all it has to respect and value the people, the language, the culture, and then it has to be equipped to be responsive to whatever needs that exist.”	“I think we need trauma-informed services and support, which means we are grounding our supports in a non-judgmental, non-oppressive, non-violent approach, [encouraging] empowerment, choice and safety.” “Trauma-informed care asks what happened to you rather than what’s wrong with you.”

**Table 4:** Foster cross-sectoral engagement and collaboration (Theme 3)

Cross-Sectoral Engagement and Collaboration			
**Wrap-around Care**	**Social Determinants of Health**	**Collaboration**	**Education**
• Current services often operate in silos and do not address the holistic nature of health and wellness. More wrap-around services that are family- or individual-oriented and comprehensive are needed across sectors; and services in which a number of organizations work together to provide a holistic program of supports and services for Indigenous clients	• Advocate for ‘whole health’ – food security, shelter, safety, community and social support, and access to health services. This requires all the sectors to work together • Meeting the basic needs of Indigenous peoples first, such as housing and access to affordable foods	• Interagency collaboration can support delivery of wrap around services and programs • Consistent and ongoing collaboration required across the sectors (health, education, justice) • Break down the silos between the sectors by creating a safe space for decision-makers and providers across the sectors to come together to coordinate services and delivery	• Decolonize the sectors by promoting understanding and awareness of past historical trauma • A restorative justice remedy is one that places the emphasis on healing the harm done by the offence and rehabilitating the offender to avoid future harms. Such processes are in line with traditional Indigenous views of justice and healing
**Participant Quotes**			
“…it’s about sectors working together, it’s about collaboration and more wraparound services between sectors. So, justice, health, education, community supports coming together.” “Like collaboration is necessary for wraparound services to happen. But it’s more than collaboration, like, you know, is it a formal team approach. Is it where we have a judge let’s say, who is hearing a case, can consult with a mental health professional to say, this is the case that I'm dealing with, you know, to understand the realities the individual is faced with.” “So it’s about systems thinking, expanded thinking, to see the full picture and how all the systems such as education, health, justice, etc. function…and working with allies across the systems to provide more wraparound service.”	“So what are the adversities the family is experiencing? Is it historical? Is it intergenerational trauma? Is it residential school? What might contribute to that? And then how can we support it? […] knowing what these individual needs are, and then [in] the community? […] Where are the gaps? And then how do we bring them all together? So not just looking at health, education, or justice, but the whole facet of it.”	“I think an important first step is the openness to explore within each sector, own biases, belief systems, and prejudices that underlie how we provide services.” “Between the different sectors we can create a board to facilitate or co-facilitate the supports for our clients. Because a lot of times we serve the same clients.” “We need to start to think outside the box and figure out ways to navigate these systems that are kind of making us feel a little bit inside a box…we need to be open to offering services in different ways.” “It’s being able to trust the other agencies and work together collaboratively to communicate, so that in times of need or when you need that information, you can reach out to other services, like Child Services and say hey, this is what I need. But in order for that to happen, there has to be a trust in the relationship.”	“I think it’s really important to bring back what was lost. But it’s also difficult for Elders and Indigenous health workers to enter into institutions or organizations that have been, were colonial” “Sectors have a responsibility to spend more time actually learning and understanding the history [of Indigenous peoples].”

**Table 5:** Focus on children and youth (Theme 4)

Children and Youth			
**Early Investments (Preconception to early childhood)**	**Engaging Parents and Families in the Schools**	**Culturally Appropriate Counselling for Youth**	**Mentorship**
• Foster healthy childhood development which begins during pregnancy and infancy	• Engage the parents in the school and in their child’s learning • Involve Elders as mentors in the schools to support Indigenous school-aged children	• Focus on specialized counselling services for children and youth in the schools and in communities	• Train youth to be peer-mentors in the schools or in their communities
**Participant Quotes**			
“We talk about supporting early childhood development…[but] we forget that their brains are changing [like] rapid fire in their teen years…It’s really important that we sustain their development throughout the years and bring in the knowledge and expertise of Elders in the school to support them [youth]…we need to provide supports throughout the lifespan.” “So when you experience relationship violence or gender-based violence…and we know that if youth are experiencing violence then they are likely going to continue that violent behavior when they’re older. So we need to do a better job at intervening and creating supports and interventions so that youth who experience violence or trauma within personal relationships are not going to go on and continue those cycles of violence later in life.” “Education must start at home. Families, teach their children the culture, how to live, how to respect, how to treat others.”	“It’s going back to that holistic model of health…we need to start with the families and the parents, that where you can do the best prevention and to create healthy children. It has to start in our communities and our homes, because that’s where the wisdom and the knowledge is.”	“We don’t have enough pediatric and youth mental health services, we simply don’t…it’s a greater challenge to get them to come to the rural areas, especially those specialized services”	

#### Ethics

Before the forum commenced, participants provided written informed consent to take part in the audio-recorded roundtable discussions and community visioning process. Ethical approval was granted by University of Alberta’s Research Ethics Board (Pro00070845).

## Results

### Priorities and directions for Indigenous mental health

Four themes emerged for setting priorities and directions for Indigenous mental health: a) understand the realities of mental health experience for Indigenous peoples, b) design a holistic and culturally rooted mental health system, c) foster cross-sectoral engagement and collaboration, and d) focus on the mental health of children and youth. The themes are presented below with a short description. Example quotes are presented in Tables 2, 3, 4, 5.

#### Theme 1: Understand the realities of Indigenous mental health experience

Several key concepts from the roundtable discussions led to a better understanding about the realities of mental health experiences among Indigenous peoples. These concepts included gaps in mental health services, the need for provider education, a focus on destigmatizing mental health, and the inclusion of Indigenous voices in the design and delivery of mental health services. To address the gaps in mental health services, participants highlighted the importance of attention to access of mental health services or supports for Indigenous peoples. Gaps to be addressed included the need for increased frequency, consistency, and availability of mental health services in rural communities; improved transportation to access services; offering of services in Indigenous languages; and reducing system barriers by providing additional funding for mental health professionals to increase time spent in communities and with clients in rural and remote settings. For education, it was established that it is critical for mental health providers to understand the local context and experience of Indigenous peoples, to have knowledge and awareness about the history of Indigenous peoples in Canada and the lasting impacts of colonialism and intergenerational trauma, and to understand the social determinants specific to Indigenous peoples that play an important role in their mental health. Participants emphasized that this requires more than just cultural sensitivity training or certification. It involves changing how care is provided at the institutional level to provide supportive and safe environments for Indigenous clients. Tied to the gap in funding for providers, the need for sufficient time with Indigenous clients was discussed as a component of building trusting provider–client relationships and allowing providers to gain a deep understanding of the root social causes of health inequities.

To address destigmatizing mental health, participants suggested changing the way it is discussed, with positive framing and emphasizing health rather than illness. The idea of removing stigma and empowering individuals to understand mental health and wellness was also introduced. The inclusion of Indigenous voices in the design and delivery of services was highlighted by participants and in particular, engagement and involvement of Indigenous Elders in the design and delivery of services. Also emphasized by participants was the importance for providers and government to understand what healing and wellness means to Indigenous peoples, and for communities to drive the focus of Indigenous-led mental health programing and service delivery.

#### Theme 2: Design a holistic and culturally rooted mental health system

The key concepts under this theme focused on the inclusion of Indigenous peoples in decision-making on mental health service delivery, as well as harnessing resources within local communities using a strengths-based approach. In addition, providing culturally appropriate and trauma-informed care was highlighted as critical for mental health service provision in the region. Allocating funding to train and hire Indigenous mental health workers from the communities was suggested as this could build local capacity and provide cultural support and traditional healing practices to clients while encouraging programs and services to focus on strengths that are already within communities. Furthermore, including traditional knowledge in the delivery of mental health services was deemed necessary for supporting culturally appropriate care. Participants also spoke about promoting culturally strengthening factors, such as connection to family and kinship and “bringing back what has been lost” as a result of dominant western approaches to treatment such as traditional healing practices, Indigenous language, and Indigenous knowledge. Multiple areas for Elders or Knowledge Keepers to be engaged in as ‘cultural mentors’ were identified including in the training of mental health professionals, in schools to teach youth about mental wellness through cultural and traditional practices, and in paid positions working alongside western health providers for home visits. Moving away from the medical model of sickness and instead including Indigenous knowledge frameworks, such as the Medicine Wheel, was recommended for a holistic approach to healing that is focused on the ‘whole person’. One participant shared that if we are not open to new ways of doing things, then the mental health system and people working within the system “will remain stuck in this perpetual state of doing the same thing over and over that doesn’t work.” Participants also explained that the way the mental health system works now, with mental health professionals using individualized approaches and solutions, is not aligned with an Indigenous holistic view of health and wellness. The holistic approach would take into consideration the individual, family, and community needs to promote healing and recovery. Additionally, the need for cultural sensitivity across the spectrum of mental health care, from prevention to promotion, was also identified. When discussing trauma-informed care, the embedding of Indigenous culture and values into treatment and healing was described. Other recommendations included the provision of addiction and counselling supports across an individual’s lifespan, and the acknowledgment and implementation of the TRC recommendations regarding health across health service organizations.

#### Theme 3: Foster cross-sectoral engagement and collaboration

The key concepts within this theme that emerged from participant discussions included providing wraparound care, addressing the social determinants of health, collaboration between agencies and sectors, and educating across sectors. From roundtable discussions participants emphasized that the basic needs of individuals must be supported to achieve positive mental health. In order to provide comprehensive holistic support that includes meeting Indigenous peoples’ basic needs (e.g., housing, food security, shelter, safety, community, social support, health services), organizations need to regularly work collaboratively. Establishing a sustainable long-term interagency committee or board that coordinates the different sectors was suggested by participants as a way to foster collective understanding and collaboration on mental health service delivery. It was also mentioned that the sectors need to be decolonized through education regarding past historical trauma and by implementing systemic changes that prioritize anti-racism and culturally safe environments to provide mental health care. Also, participants stated that strengthening mental health services and programs for Indigenous clients, service organizations, funders, decision makers, and healthcare professionals must carefully consider the 94 Calls to Action from the TRC.

Participants also described that the justice sector has a role to play, with the suggestion for promoting restorative justice that is more in line with traditional Indigenous views of healing and rehabilitative justice. Some participants spoke about acknowledging and bringing forward Indigenous legal traditions. Indigenous legal approaches to crime and addressing harm reduction principles that can be important to promoting community healing, reconciliation, and the reintegration of the offender.

#### Theme 4: Focus on children and youth

The key concepts described by participants for child and youth mental health included making early investments in children’s health and social development, engaging parents and families, promoting youth mentorship, and providing culturally appropriate counselling services for Indigenous youth. The need for supports across the lifespan was emphasized, as supporting healthy early childhood development begins during pregnancy and infancy. Family engagement and mentorship, the inclusion of parents and families in the school and children’s learning as well as the involvement of Elders and peers as mentors were identified as key elements of healthy families and the holistic model of health and well-being [67]. Indigenous peoples believe caring for children is the shared responsibility of their communities. Therefore, participants described the importance that programs or services honor traditional beliefs of well-being and parenting practices. Some participants went on to share that a traditional way of life taught children the importance of connectedness to family and their nation, whereas contemporary society and the education system teaches about isolation and individual gain. This lost sense of connection and belonging has then resulted in a loss of purpose, pride, and identity. When considering existing gaps in the mental health system, participants noted that there is a lack of specialized care and supports for children and youth. Thus, dedicated and specialized mental health services for Indigenous children and youth was considered critical, and should have all sectors (health, education, community and justice) working together.

#### Community visioning results

From the participatory exercise on day two of the forum, a vision for strengthening Indigenous mental health was formed (see Additional files 1 and 2 for graphic recordings of the community vision). The community vision created by participants included: core guiding principles; taking a strengths-based perspective; respecting Indigenous knowledge; and breaking down the stigma of mental health. There was consensus that the design and delivery of mental health services should take a strengths-based approach to promote mental wellness among Indigenous residents and communities in the region. Focusing on the positive was also discussed in the context of existing data on mental health outcomes in Indigenous populations that is deficit-focused. Additionally, participants voiced that Indigenous-led health partnerships and services need to be grounded in traditional Indigenous knowledge — upheld by community Elders— rather than being grounded in western medicine, structures, and knowledge.

## Discussion

The two-day Indigenous mental health forum led to the identification of priorities and directions for Indigenous mental health in the northern region of Alberta, Canada. Researchers, community leaders, service providers, and decision-makers across the sectors gathered to build a shared vision for strengthening mental health services and programs for the region’s diverse Indigenous population. Participants envisioned a holistic and integrated mental health model that is Indigenous-led, culturally based, and blends western modalities with Indigenous knowledge and healing practices [24, 68]. Given the importance of traditional healing practices and culturally appropriate care for Indigenous people, it is critical that these sources of healing are integrated into their care. Furthermore, an integrated care model utilizes the strengths and collaborative skills of many health professionals and specialists, as well as collaboration with community, families, and caregivers [69]. Enrlich and colleagues (2015) engaged multiple stakeholders across sectors within the health system in Australia to identify a range of collaborative actions and processes to improve the physical health of people with severe mental illness [70]. The findings from this study also reinforced the need for holistic care and the integration of health services.

Despite evidence supporting integrated models for mental health service delivery for Indigenous peoples, challenges with implementing integrated models of care involving cross-cultural therapeutic cooperation have been identified. For instance, Carrie et al. (2015), conducted a policy review of Nicaraguan health plans at the national and regional level that support the integration of traditional medicine and biomedicine. The authors found that although measures to create therapeutic cooperation are woven into Nicaraguan health plans at the national and regional level, in practice, the delivery of integrated health services has been implemented with varying results.

Participants of the forum also mentioned that existing mental health services and funding should be realigned to build a more culturally based system of mental health services. With regards to having more Indigenous people educated in the mental health profession, Indigenous communities are already employing Indigenous methods and approaches and engaging Elders, Knowledge Keepers, and healers to support system transformation within the area of health [34]. The training and hiring of Indigenous people within the mental health profession to support holistic mental health service delivery was identified as a key priority by participants for supporting their vision towards Indigenous-led, culturally safe, and equitable mental healthcare in the region.

Participants advocated for holistic approaches to mental health delivery for Indigenous people, rather than individualized approaches to mental health that exclude family and kinship networks supporting the individual’s healing journey. Rather, holistic approaches to mental health need to look at the individual’s mental health state and experience within the context of their family and community structure. Therefore, family and kinship networks should be involved in the person’s healing journey. Participants further explained that the utilization of the Medicine Wheel can serve as a framework for improving the mental health and well-being of Indigenous people in the region. The Medicine Wheel is used to explain an Indigenous worldview of life that everything in life is connected. All four elements of the Medicine Wheel interact together to form a strong, healthy person. It can also be used to guide transformation of mental health services.

Championing self-determination is highlighted as an important factor in the development of Indigenous mental health services globally. In Australia, closing the mental health gap among the Aboriginal and Torres Strait Islander people is a national priority. Policy responses have advocated for self-determination to the provision of Aboriginal and Torres Strait Islander mental health services, and support for Aboriginal and Torres Strait Islander leadership in the Australian mental health system [71]. In Canada, the TRC Call to Action #22 states, “We call upon those who can effect change within the Canadian health care system to recognize the value of Aboriginal healing practices and use them in the treatment of Aboriginal patients in collaboration with Aboriginal healers and Elders where requested by Aboriginal patients” [8]. This call to action highlights the need for self-determination in use of traditional knowledge and healing practices which have persevered despite systemic oppression and colonial policies. There are several examples in Canada of Indigenous-led and Indigenous-driven mental health initiatives that promote Indigenous knowledge, culture and language. It was clearly identified during the roundtable group discussions that mental health services and supports must be driven by the needs and priorities identified by communities. Services must also target clearly identified measures of wellness. A systematic literature review found that mental health services developed by Indigenous communities in Canada were the most effective at providing appropriate care [19]. Co-creating Indigenous models of care with Indigenous communities has also been recommended to support self-determination, increased community participation in health, and increased community capacity [68]. Maar, Erskine [22] found that a collaborative, community-based Indigenous mental health care model led to improvements in culturally-safe care for Indigenous communities in northern Ontario, Canada [22]. Furthermore, Kyoon-Achan, Philips-Beck [72] created a framework for mental wellness that was co-developed with Indigenous communities to include culturally informed approaches, allowing participants to articulate their experiences and to advocate for and support culturally informed mental health services. Therefore, there is growing evidence towards Indigenous-led healthcare partnerships for improving health outcomes and access to care for Indigenous peoples [25].

The COVID‐19 pandemic has also highlighted vulnerabilities in local workforces that are highly dependent on staff from out of community. As highlighted during the forum, long‐term initiatives to build local capacity are needed, given the significant issues which have arisen in relation to available workforce (including the need to isolate fly‐in‐fly‐out staff and local Indigenous staff who are unable to work). There is a need to ensure Indigenous health workers are supported to provide the best care for communities, especially in areas with pre-existing staff shortages. The training of Indigenous health workers will help reduce the reliance on out‐of‐community mental health practitioners providing services to remote and rural communities and more importantly, address surge workforce planning in Indigenous and rural communities in anticipation of future public health crises and the associated mental health and social impacts.

Participants at the forum highlighted that the different sectors need to be open and willing to explore different ways or models of delivering mental health services that is inclusive of different knowledge systems on health (i.e., both western and Indigenous knowledge), healing practices, and the preferences of Indigenous clients. Mental health promotion requires multi-sectoral action across health, education, community, and the justice sectors, and through the delivery of culturally safe, strengths-based, family- and community-oriented mental health and wellness programs, services and policies that aim to promote healthy emotional, spiritual, and social development in childhood and adolescence, as well as in those at risk of poor mental health [73, 74]. More importantly, mental health experiences and strengths vary among Indigenous communities and individuals in Canada and across the globe, and this variation reflects the distinctiveness of Indigenous peoples’ histories, languages, cultures, environments, and worldviews. Similar to our study, researchers in Australia worked with Indigenous peoples to create a framework of well-being priorities, which included relationships, empowerment, and culture are key priorities [75]. This framework was tested in four Indigenous remote communities. Further, the authors highlighted the importance of aligning priorities with the values of Indigenous communities and strengthening collaboration among services outside the health sector.

Decolonizing mental health is more than just practicing cultural competence — the ability to understand and interact with people of different cultures. Instead, it is recognizing that for many Indigenous peoples the trauma from oppression and colonization plays a major role in their mental health state. Stigma and discrimination remain a barrier to accessing mental health services for many Indigenous peoples in Canada and in other parts of the globe [26, 76]. The values and traditions of Indigenous persons may be poorly understood and their concepts of wellness and ways of knowing sometimes undervalued.

The cumulative stress caused by natural disasters and the current global COVID-19 pandemic is detrimental to the mental health and well-being of children, youth, and families [77]. During the current pandemic, families are experiencing stress, anxiety, concern and even fear. Unique challenges have been documented for Indigenous communities across Canada, particularly elders who are most at risk, and northern and remote communities that have limited access to services and other types of supports [78–80]. The pandemic has been reported to compound existing mental health concerns and may bring up past or lingering trauma for Indigenous peoples who have endured historical trauma from colonialist policies [37, 81, 82]. Moreover, disasters and emergencies have been found to result in some positive experiences, including strengthened relationships and social networks [77]. It is important that we highlight the strength and resilience of Indigenous peoples during challenging and traumatic events like a natural disaster or public health crisis. Research that examined how Indigenous residents coped after the 2016 Horse River wildfire revealed the importance of kinship, social and community support, and connection to culture as positive coping mechanisms and factors influencing mental health and well-being [83].

The themes that emerged from the forum offer important insights for future research, practice, and policy. Firstly, the forum was inclusive of diverse perspectives from people of Métis and First Nations ancestry, and across urban and rural community settings. Different Indigenous groups, such as Métis [19, 23] and urban or off-reserve First Nations can have unique experiences that are often excluded in research and data. Secondly, the provincial Alberta Government’s 2020–2023 fiscal plan commits $100 M for a new mental health and addiction strategy, which is intended to address the ongoing challenges of mental health and addictions in Alberta [84]. However, there is no mention of how (or if) funding for mental health services or supports will be allocated to Indigenous communities. This presents a window of opportunity to advocate for improved, equitable and culturally resonant mental health services and programs for Indigenous populations in the northern province.

### Strengths and limitations

The modified nominal group consensus method used for this research ensured equal participation among participants. The structure of the nominal consensus process maximized the uptake of expertise from participants and minimized the potential of one personal or professional perspective dominating the process. However, our study has some limitations. We had limited participation of youth (*n* = 3) at the forum. Despite recruiting through social media and connecting with representatives of youth programs offered at Indigenous organizations, we were unable to recruit a desirable number of youth to this study. Nonetheless, the study is strengthened by the diverse range of cross-sectoral service providers and community Elders. As is the case with many recruitment approaches, there was the potential that not all Indigenous language groups in the region (Cree, Dene, Michif) were adequately represented. For instance, there were more Cree-speaking Indigenous participants at the forum, in comparison to Dene-speaking or Michif-speaking Indigenous participants. A further limitation is that we restricted voting on the priorities and directions to one round and may therefore have limited the level of consensus achieved.

## Conclusion

This study illustrates that collaborative and consensus-based facilitation approaches are effective methods for prioritizing community-based knowledge and expertise on setting priorities and directions for an Indigenous mental health strategy in the RMWB. The forum was also a first step towards fostering multi-sectoral and community relationship-building and collaboration on Indigenous mental health. This study makes an important contribution to improving Indigenous mental health through cross-sectoral engagement. Forum participants emphasized that Indigenous-focused services foster relationships and collaboration between care providers, communities, families, and caregivers [69]. Moreover, Indigenous-focused services ensure Indigenous knowledge, local context, access, and integration of services is supported. The Indigenous mental health forum allowed for a better understanding of the experiences of Indigenous people in the RMWB with accessing mental health services, as well as the Indigenous mental health needs and directions for improving Indigenous mental health service delivery in the RMWB. These outcomes of the modified NGT process, modified Dotmocracy method, and community visioning exercise provided important contextual information and fostered collective action for shaping future responses on Indigenous mental health in region.

## Supplementary Information

**Additional file 1. **

**Additional file 2.**

## Data Availability

Data sharing is not applicable to this article as no datasets were generated or analyzed during the current study. All qualitative data analysis is presented in Tables 2, 3, 4, 5, and transcriptions of roundtable group discussions are stored in a secured drive on a password protected computer.

## Abbreviations

RMWB: Regional Municipality of Wood Buffalo
MNA: Métis Nation of Alberta
TRC: Truth and Reconciliation Commission
AHS: Alberta Health Services
CBPR: Community-Based Participatory Research
