# Supplementary figures and images for: Identifying priorities, directions and a vision for Indigenous mental health using a collaborative and consensus-based facilitation approach
Source: BMC Health Serv Res. 2022 Mar 27;22:406. doi: 10.1186/s12913-022-07682-3 (PMC8958486; doi:10.1186/s12913-022-07682-3)

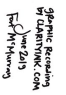

Supplement: Supplementary file 1 — Additional file 1. [file 12913_2022_7682_MOESM1_ESM.pdf]
