# Supplementary material for: Identifying priorities, directions and a vision for Indigenous mental health using a collaborative and consensus-based facilitation approach
Source: BMC Health Serv Res. 2022 Mar 27;22:406. doi: 10.1186/s12913-022-07682-3 (PMC8958486; doi:10.1186/s12913-022-07682-3)
Supplement: Supplementary file 2 — Additional file 2. [file 12913_2022_7682_MOESM2_ESM.pdf]

MOVING FORWARD TOGETHER

SUPPORTING INDIGENOUS LED, CULTURALLY SAFE AND EQUITABLE MENTAL HEALTH CARE ACCESS

STRENGTHS

AWARENESS THAT MENTAL HEALTH AFFECTS THE WHOLE BODY

DESIRE FOR CHANGE

WE WORK WELL TOGETHER

BRING BACK THE MEDICINE WHEEL

COMMUNITIES ARE READY FOR CHANGE

STRONG PARTNERSHIPS IN RURAL COMMUNITIES ARE ALREADY IN PLACE

ELDERS AND TRADITIONAL MEDICINE

LET OUR VOICES BE HEARD

ENTHUSIASM

PASSIONATE

COLLECTIVE ENERGY OF THE GROUP

MOTIVATED STAKEHOLDERS and KNOWLEDGE KEEPERS

ACTION PLAN

CREATE AN ADVISORY COMMITTEE SPECIFIC TO INDIGENOUS MENTAL HEALTH

ENGAGE SERVICE PROVIDERS, WITH SUPPORT OF CULTURAL LEADERS TO DELIVER OTHER PROGRAMMING AT HEALTH CENTRES TO END STIGMA OF GOING TO COUNSELLING

TRAINING FOR NON-INDIGENOUS TO BE IN A BETTER POSITION TO HELP

SPECIFIC PSYCHIATRIC PROFESSIONALS FOR BOTH ELDERS AND CHILDREN
- currently no paediatric psychiatrists

USE THE INFORMATION PUT IT INTO ACTION AND INVOLVE AGENCIES, PROVINCE and FED. GOVT.

HAVE THEM ASSIST WITH FUNDING AND ALLOCATE to each community involved in this meeting

ESTABLISH A HEALTH ADVISORY TO PRESENT TO FEDERAL HEALTH SERVICES

SHARE INFORMATION WITH HEALTH CARE MGRS RESPONSIBLE FOR CARING FOR INDIGENOUS COMMUNITIES:

- AHS PUBLIC HEALTH - JANVIER
  FT. McMURRAY FIRST NATION
- FORT McKAY FIRST NATION HEALTH CENTRE
- FIRST NATION'S INUIT HEALTH
- CHIPEWYAN PRAIRIE FIRST NATION HEALTH CARE CENTRE

CONNECT WITH THE AHS 'INDIGENOUS STRATEGY' LEADERSHIP

IN PERSON RECONNECTION / FOLLOWUPS SCHEDULED TO MAINTAIN MOMENTUM
- include tele/video for those who can't attend.

PROVIDE INTERPRETATION SERVICES

WORK FOR THE PEOPLE NOT AGAINST THE PEOPLE

FORM AN INDIGENOUS/METIS/FIRST NATION HEALTH COMMITTEE WITH REPRESENTATION FROM ALL COMMUNITIES
- Become go to people who take information to AHS

COMMUNICATION BETWEEN ORGANIZATIONS and KNOWLEDGE

MEET WITH CHIEF and COUNCIL TO SHARE THIS VISION

ENSURE THAT RURAL COMMUNITIES ARE INCLUDED IN DECISION-MAKING

"GET TO KNOW YOU" EVENT. INVITE SERVICES AND THE PUBLIC

PROPOSAL REQUESTING FUNDING FROM INDUSTRY FOR DEVELOPMENT OF STAND ALONE RESOURCE PLACE
- ONE STOP SERVICES

ACCESS TO SUPPORT INSIDE THE JUSTICE SYSTEM FOR THOSE AFFECTED BY INCARCERATION

graphic Recording by Lori - clarityink.com
JUNE 2019
Fort McMurray

McMURRAY MÉTIS
UNIVERSITY OF ALBERTA SCHOOL OF PUBLIC HEALTH
